# Mapping Genetic Events of SARS-CoV-2 Variants

**DOI:** 10.3389/fmicb.2022.890590

**Published:** 2022-07-14

**Authors:** Luyao Qin, Jing Meng, Xiao Ding, Taijiao Jiang

**Affiliations:** ^1^Institute of Systems Medicine, Chinese Academy of Medical Sciences, Peking Union Medical College, Beijing, China; ^2^Suzhou Institute of Systems Medicine, Suzhou, China; ^3^Institute of Basic Medical Sciences Chinese Academy of Medical Sciences, School of Basic Medicine Peking Union Medical College, Beijing, China; ^4^Guangzhou Laboratory, Guangzhou, China

**Keywords:** SARS-CoV-2, genetic events, genetic map, mutation and recombination, evolutionary pathway

## Abstract

Genetic mutation and recombination are driving the evolution of SARS-CoV-2, leaving many genetic imprints which could be utilized to track the evolutionary pathway of SARS-CoV-2 and explore the relationships among variants. Here, we constructed a complete genetic map, showing the explicit evolutionary relationship among all SARS-CoV-2 variants including 58 groups and 46 recombination types identified from 3,392,553 sequences, which enables us to keep well informed of the evolution of SARS-CoV-2 and quickly determine the parents of novel variants. We found that the 5′ and 3′ of the spike and nucleoprotein genes have high frequencies to form the recombination junctions and that the RBD region in S gene is always exchanged as a whole. Although these recombinants did not show advantages in community transmission, it is necessary to keep a wary eye on the novel genetic events, in particular, the mutants with mutations on spike and recombinants with exchanged moieties on spike gene.

## Introduction

A newly emerged betacoronavirus named severe acute respiratory syndrome coronavirus 2 (SARS-CoV-2) has been ongoing in the world and evolved continuously (Wu et al., 2020a; Plante et al., 2021; Wu et al., 2021). As an RNA virus, genetic mutations play a central role in the evolution of SARS-CoV-2, including the substitution of a single nucleotide, deletion, and insertion, which result mainly from replication errors, base editing, and nucleic acid damage (Sanjuán and Domingo-Calap, 2016). Genetic analyses showed that more than 29,000 single-nucleotide polymorphisms (SNPs) and over 10,000 insertion/deletions have been detected in the SARS-CoV-2 genomes in nearly 2 years^1^. The high mutation rate, which was estimated to be 10^−3^ substitutions per year per site (Bar-On et al., 2020), led to a high genetic diversity of SARS-CoV-2. The adaptative advantages of different mutations play an important role in natural selection during the evolution of SARS-CoV-2. Velazquez-Salinas et al. indicated the positive selection at specific residues of the accessory proteins ORF3a and ORF8, which drove the early evolutionary trends of SARS-CoV-2, and explored the importance of epistatic interactions among sites in the generation of variants adapted to humans (Velazquez-Salinas et al., 2020). Subsequently, the World Health Organization (WHO) defined some variants as the Variants of Concern (VOC) including Alpha, Beta, Gamma, Delta, Omicron, Variants of Interest (VOI) including Lambda and Mu, and Variants Under Monitoring (VUM). Our previous work showed that some mutations occurred simultaneously, forming co-mutation modules in the genomes of SARS-CoV-2. By focusing on the co-mutated nucleotides, we classified the SARS-CoV-2 population into different groups, each corresponding to a genotype with a set of co-mutations (Qin et al., 2021).

Mutations result in genetic diversity by changing the nucleotides in specific positions. Recombination shuffles these mutations by exchanging genetic materials to further increase the genetic diversity (Arenas et al., 2018). In most cases, recombinations are caused by the viral polymerase of active replication jumping from one template to another, which provide the viruses with the ability to better adapt to current hosts or to infect new hosts (White et al., 2011). The large SARS-CoV-2 RNA genomes allow genome modifications caused by recombinations (Su et al., 2016; Jungreis et al., 2021), where co-infection is the prerequisite of recombination for SARS-CoV-2. Co-infection provides an opportunity to exchange gene fragments when at least two genetically distinct genomes are within the same host cells (King et al., 1982). With the co-circulation of multiple SARS-CoV-2 variants, more and more evidence showed that co-infection events have occurred in individuals, leading to genetic recombinations (Hashim et al., 2020; Varabyou et al., 2021; Zhou et al., 2021). A recent study indicated that the Alpha variant was involved in multiple recombination events, where some recombinants inherited the S gene from Alpha (Jackson et al., 2021). David VanInsberghe et al. identified five recombinant SARS-CoV-2 genomes as of August 2020 (VanInsberghe et al., 2021), and Ales Varabyou et al. detected 225 likely recombinants from 87,695 genomes. These studies revealed an obvious signal of genetic recombinations in SARS-CoV-2 (Varabyou et al., 2021).

The above studies showed that the genetic mutations and recombinations have occurred in SARS-CoV-2 frequently and left many genetic imprints in the SARS-CoV-2 genomes (Lam, 2020). Although some mutants and recombinants have been reported in some studies, there is no work to identify all the variants in SARS-CoV-2 and the evolutionary relationships between them. With the increase of high-quality genomic data, it is feasible to depict the evolution pathway, which facilitates the prevention and control of SARS-CoV-2.

In this work, we constructed a complete genetic map showing the explicit evolutionary relationships among all SARS-CoV-2 variants based on the genetic imprints, which enables us to track the evolutionary pathway of the novel variants quickly. As of 31st October 2021, we identified the genetic events of all the downloaded SARS-CoV-2 genomes, where 58 groups involving genetic mutation and 46 recombination types including 1,229 recombinants were identified. For these recombinants, the spatio-temporal distributions showed the co-circulation of their parents and indicated that these recombinants did not have advantages in community transmission. We found that SARS-CoV-2 had a high frequency to form recombination junctions in the 5′ and 3′ of S gene and N gene. Most notably, the receptor-binding domain (RBD) of S gene is always exchanged as a whole, which may be associated with the observation that the current recombinants did not develop into dominant variants. In summary, we developed a novel method to identify the genetic events, including genetic mutation and recombination, of SARS-CoV-2 variants to track its evolutionary dynamics, where the characteristics of identified genetic recombination events were further analyzed.

## Materials and Methods

### Data Collection and Processing

A total of 3,392,553 SARS-CoV-2 high-quality genomes sampled from humans were downloaded from the GISAD database as of 31st October 2021 with the labels of “complete,” “high coverage,” and “collection date complete.” 2,817,027 sequences submitted to GISAD as of 30th September 2021 were used to identify the co-mutations. After the data processing using the pipeline described in the previous work, the remaining 2,454,712 genomes were used for the genetic grouping. The 3,392,553 genomes were aligned to the SARS-CoV-2 reference genome in GenBank (NCBI Accession number: NC_045512.2; Wu et al., 2020b) using MAFFT (Katoh et al., 2002) and were analyzed in the following work.

### Identification of Genetic Events

#### Labeling Co-mutations in the Genome

Our previous work classified the SARS-CoV-2 population into different groups based on the co-mutation modules instead of the phylogenetic tree. Each group corresponds to a set of specific co-mutations that captured the vital evolutionary information of SARS-CoV-2 and the evolutionary relationship between groups accurately. In summary, 58 groups involving 247 co-mutations were identified. In this work, we used specific co-mutations in different groups and the association of mutation and recombination to identify genetic events in SARS-CoV-2 genomes. For each SARS-CoV-2 sequence, we searched for these co-mutations in the genome and labeled them with the corresponding group name, such as “G3, G3, G3.2.6, G3.2.6. G3.14, G3, G3.14.1, G3.14.1.” The label would be changed into the adjacent label when the neighboring group name is its sub-group. For example, “G3, G3.2.6” would be changed into “G3.2.6, G3.2.6.” After the iteration, each sequence is represented by a set of mutually exclusive group names sorted according to the position of the mutation site, such as “G3.2.6, G3.2.6, G3.2.6, G3.2.6, G3.14.1, G3.14.1, G3.14.1, G3.14.1.”

#### Determining the Genetic Events

Based on the source and distribution of these co-mutations in the SARS-CoV-2 genome, the genetic event could be determined. First of all, the genome was considered as G0 if it did not contain any co-mutations. In addition to G0, there were three cases. Case 1: all the co-mutations were from the same group, indicating that the virus belonged to this group. Case 2: the co-mutations detected in the genome were from two or more groups. In the label set, the distribution of these group names was irregular, that is, the same group names did not form the block structure. Then, the virus was considered as a mutant, which has the largest number of identical co-mutations with its parental group, compared with the other groups. Case 3: the detected co-mutations were from two or more groups, and the same group names formed a block structure, which was regarded as the genetic recombination. Here, we only detected the recombination events hosted by two parental groups. In this way, not only the genetic events in the virus have been identified, but also the parents of the virus have been pointed out.

### Validation of the Detected Recombination Events

#### Verifying the Parental Groups

To verify the parental groups, we first searched for the direct ancestor of each recombinant fragment from all the downloaded genomes. The sequence which was sampled from the same country at an earlier time with the highest similarity was regarded as the direct ancestor of the recombinant fragment. The inferred parental group was verified if the searched direct ancestors belonged to this group.

#### Constructing Phylogenetic Trees

The phylogenetic trees were constructed to observe the topology. The parental sequences, collected from the same country with the recombinants and collected before the recombinants in the same month, were selected. The recombinant sequences and the representative sequences in the parental groups were used to construct the phylogenetic trees of the recombinant region and non-recombinant region. The breakpoint positions inferred by RDP4 and Simplot (Lole et al., 1999; Martin et al., 2015) were different but both were located in a region where the gene exchange might occur. We first adopt the breakpoints inferred by RDP4 for analysis. The breakpoints inferred by Simplot were accepted when there is no recombination signal in RDP4. These trees were constructed with FastTree (Price et al., 2009) and visualized with FigTree (http://tree.bio.ed.ac.uk/software/figtree/).

#### Checking the Spatial–Temporal Distribution

All the sequences in the parental groups were used for the analysis of spatial distribution, while the sequences collected from the countries where the recombinants were collected were used to analyze the temporal distribution. The temporal distribution data were analyzed by EXCEL. The spatial distribution map was drawn with self-written code by calling the package “Pyecharts” of Python3.

## Results

### Framework to Identify the Genetic Events in SARS-CoV-2

**Figure 1:**
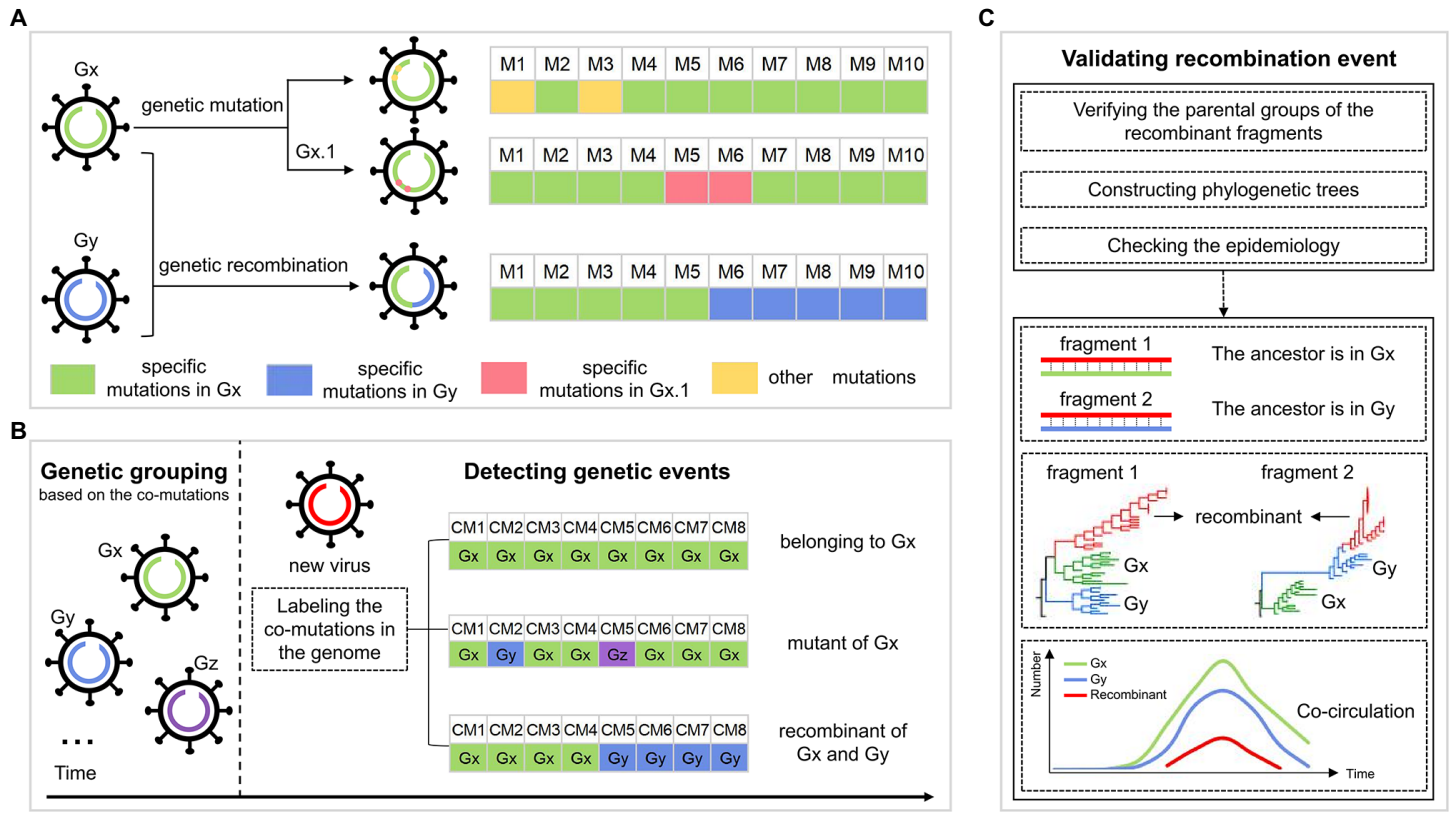
The workflow for identifying the genetic events during SARS-CoV-2 evolution. **(A)** The possible evolutionary pathways of SARS-CoV-2 viruses. Gx and Gy are two genotypes, each corresponding to a set of specific mutations, whose genomes are colored green and blue, respectively. Viruses in each genotype could evolve in two ways, including genetic mutation and genetic recombination. For genetic mutation, the descendant inheriting the specific mutations of its parental genotype has some new mutations that may be rare, or may be dominant to form a sub-genotype Gx.1. For genetic recombination, the descendant (virus with the green and blue genome) inherits mutations from the parents Gx and Gy, leading to the structure of the crossover site. **(B)** Detection of the genetic events in the SARS-CoV-2 genomes. The SARS-CoV-2 population is first classified into multiple groups, such as Gx, Gy, and Gz, each corresponding to a genotype with a set of specific co-mutations. For a new virus (virus with red genome), we label the co-mutations (CM) occurring in its genome as the corresponding group. There are three cases: (i) all the co-mutations are labeled as the same group “Gx,” indicating that the virus belongs to Gx; (ii) the largest number of the detected co-mutations come from Gx, and co-mutations from other groups are sporadic and are distributed irregularly, indicating that the virus is a mutant of Gx; (iii) the co-mutations come from different groups (Gx and Gy), where “Gx” and “Gy” both form the block structure, showing that the virus is a recombinant of Gx and Gy. **(C)** Validation of the detected recombination events. Validation is done in three ways: (i) verification of the parental groups of recombinant fragments; (ii) the topology of phylogenetic trees of recombinant region and non-recombinant region; and (iii) the spatial–temporal distribution of the parental groups.

Figure 1A shows the hypothesis about the evolution of SARS-CoV-2. Specifically, the descendants involving genetic mutations experience gene drift when inheriting the genetic characteristics of their parents, where the mutations may be rare, or may be dominant to form a sub-genotype. The recombinant inherits genetic characteristics from at least two genetically different parents and forms the structure of the crossover site. Based on the hypothesis, we developed a pipeline to identify the genetic imprints in the SARS-CoV-2 genome to figure out its evolutionary path. This work was based on the identified co-mutations from SARS-CoV-2 genomes with the method in our previous study, where the SARS-CoV-2 population was classified into multiple groups, each representing a genotype with a set of specific co-mutations (Qin et al., 2021). For an emerging SARS-CoV-2 strain, it may belong to one of the three cases according to the distribution of group-specific co-mutations. As shown in Figure 1B, case one is that all the co-mutations in the genome come from the same group, showing the virus belongs to the group; case two is that the detected co-mutations are from two or more groups and are distributed irregularly, indicating the virus is a mutant, which has the largest number of identical co-mutations with its parental group, compared with the other groups; case three is that the co-mutations are from two or more groups, and that the co-mutations with the same group form the block structure, which is considered as the genetic recombination. For the detected recombinants, they would be validated based on the similarity of these fragments with the inferred parents, phylogenetic trees, and epidemiology, which is described in Methods in detail (Figure 1C).

### The Genetic Map of SARS-CoV-2 Variants

**Figure 2:**
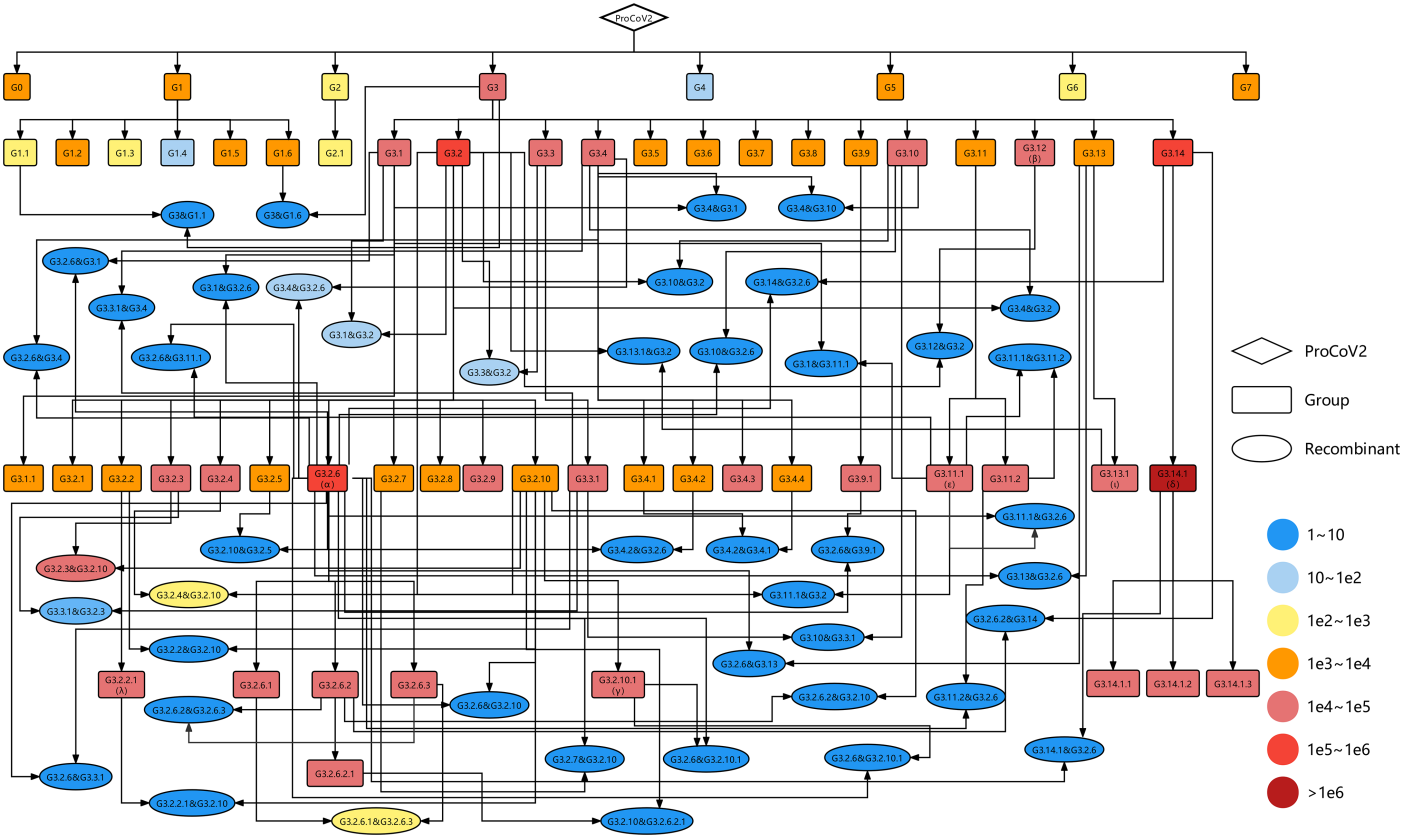
Genetic map including all identified SARS-CoV-2 variants. The diamond, rectangles, and ellipses represent the origin of SARS-CoV-2 (ProCoV), 58 groups and 46 recombination types, respectively. The recombination types were named parent 1 and parent 2, where the former is the parent of the non-recombinant region and the latter is the parent of the recombinant region. The color corresponds to the number of samples in each variant. The black lines with arrows indicate the evolutionary direction.

As of 30th September 2021, 58 groups were identified from 2,454,712 high-quality SARS-CoV-2 genomes, involving 247 co-mutations (more information shown in http://cmmgroup.grmh-gdl.cn:20023). Further, we identified the genetic events in SARS-CoV-2 genomes collected before 31st October 2021 based on the developed pipeline (Figure 1). As shown in Figure 2 and Supplementary Figure S1, 6,059 genomes did not have any co-mutations and were assigned to G0; 1,241,614 genomes were identified as case one, which contained a single source of co-mutations; 2,143,651 genomes were classified into case two, which were the mutants of groups; and 1,229 genomes were identified as genetic recombinants, in which 843 recombinants were intragenic recombinations, the others were intergenic recombinations (Supplementary Table S3). These results showed that about 41% and 27% of sequences were the mutants of G3.14.1 (corresponding to the Alpha variant) and G3.2.6 (corresponding to the Delta variant), or belonged to them, respectively, indicating that the co-mutations of these two groups were the dominant imprints in the evolution of SARS-CoV-2. In addition, the detected 1,229 recombinants were divided into 46 recombination types. These results showed that genetic mutation and genetic recombination have occurred frequently in the evolution of SARS-CoV-2.

To visualize the evolution of SARS-CoV-2, a complete evolutionary map was constructed, in which the genetic relationships among these variants involving 58 groups and 46 recombination types were shown explicitly. As shown in Figure 2, most of the groups were the descendants of G3, carrying the co-mutations C241T in 5’UTR, C3037T, and C14408T (corresponding to amino acid substitution P4715L) in ORF1ab, and A23403G (corresponding to amino acid substitution D614G) in spike protein. The Alpha, Beta, Gamma, and Delta variants in VOC and Epsilon, Iota, and Lambda variants in VOI correspond to the group of G3.2.6, G3.12, G3.2.10.1, G3.14.1 and G3.11.1, G3.13.1, and G3.2.2.1, respectively. The G3.2.6 (corresponding to the Alpha variant) and G3.14.1 (corresponding to the Delta variant) groups both evolved into three novel sub-groups, which were named G3.2.6.1, G3.2.6.2, and G3.2.6.3 and G3.14.1.1, G3.14.1.2, and G3.14.1.3, respectively. In addition, all of the VOCs and VOIs mentioned above harbored the genetic recombination events, in which the G3.2.6 group was the most common genomic donor. These results are expected since there are more opportunities for mutation and recombination in the dominant variants.

### Recombination Events Hosted by G3.2.6/Alpha and G3.14.1/Delta

**Figure 3:**
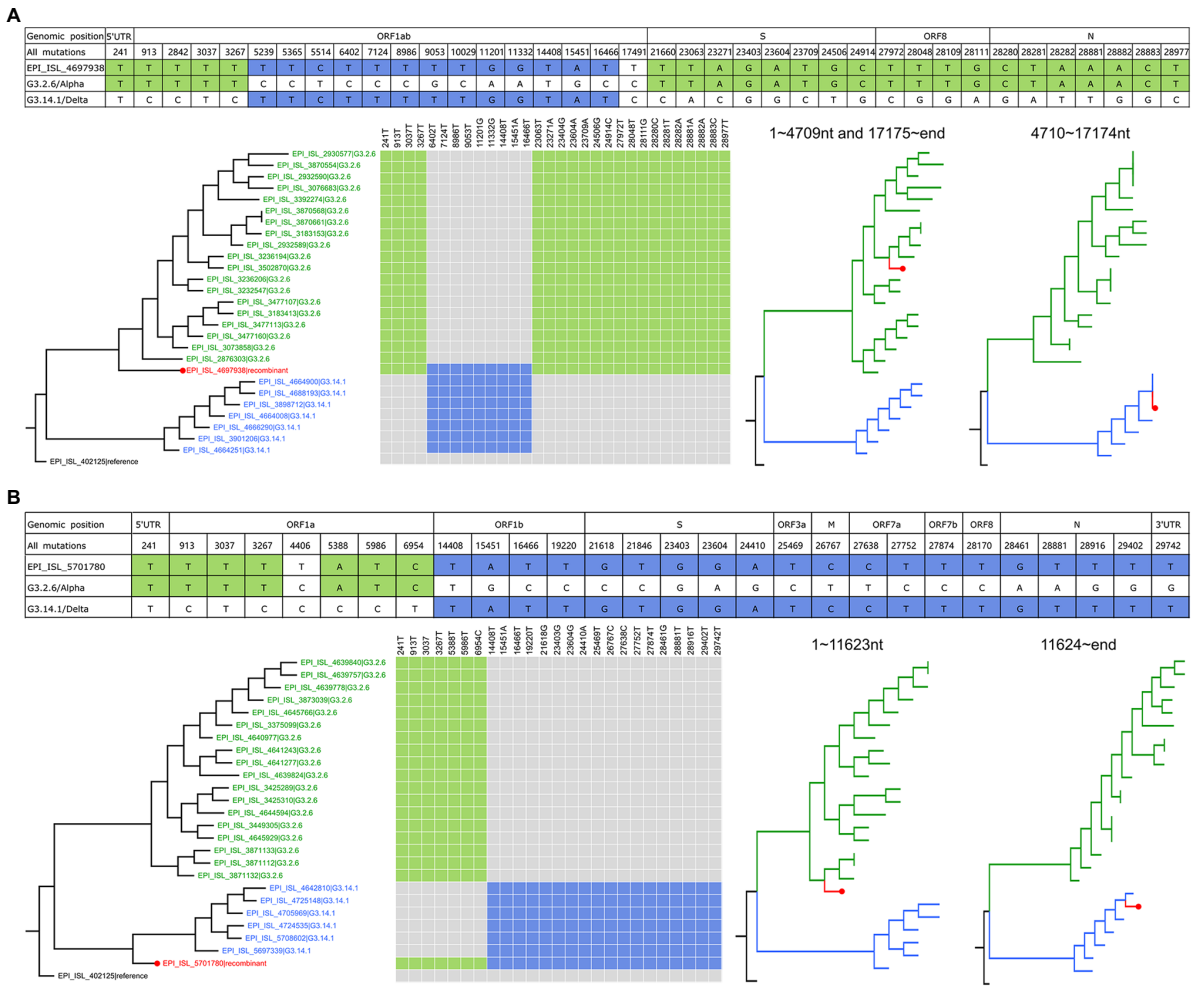
The recombination events hosted by G3.2.6/Alpha and G3.14.1/Delta. **(A)** Genetic recombination in EPI_ISL_4697938. All the mutations of EPI_ISL_4697938 and their positions in the genome are listed, in which nucleotides same with G3.2.6 are shaded in green, and nucleotides same with G3.14.1 are shaded in blue. The phylogenetic tree of whole genomes including EPI_ISL_4697938 and the representative parental sequences is on the left. The co-mutations in the recombinant genome are marked with color and are mapped on the parental sequences. The phylogenetic trees of the recombinant region and non-recombinant region are on the right. **(B)** Genetic recombination in EPI_ISL_5701780. Refer to the legends in **(A)**.

In the identified recombination events, two of them were hosted by G3.2.6 (corresponding to the Alpha variant) and G3.14.1 (corresponding to the Delta variant). As shown in Figure 3A, the genome (GISAID ID: EPI_ISL_4697938, sampled on 19th July 2021 from Japan) has the specific co-mutations of G3.2.6 in ORF1ab 5’end, S, ORF8, and N, and with the specific co-mutations of G3.14.1 in ORF1ab. It formed a structure of the crossover site and corresponded to case three. In addition, the source of all the mutations in the genome of EPI_ISL_4697938 showed the same mosaic structure, revealing that EPI_ISL_4697938 was a recombinant involving the G3.2.6 and G3.14.1. The breakpoints of this recombinant were inferred using RDP4 (Martin et al., 2015): (i) positions 1 to 4,709 and 17,175 to the end and (ii) positions 4,710 to 17,174 nt, indicating the recombination event was intragenic recombination (Supplementary Figures S4, S5 and Supplementary Table S1). Two phylogenetic trees of the recombinant region and the non-recombinant region were constructed, including the recombinant sequence and the representative sequences of G3.2.6 and G3.14.1. The result showed that EPI_ISL_4697938 clustered with the G3.2.6 variants in the phylogenetic tree of non-recombination region, and clustered with the G3.14.1 variants in the phylogenetic tree of the recombinant region. The phylogenetic trees including more information are shown in Supplementary Figures S2, S3. Besides, the sequence similarity networks visualized by Simplot++ (Samson et al., 2022) showed that EPI_ISL_4697938 was more similar with G3.14.1 in 4710 ~ 17,174 nt of viruses genomes than that with G3.2.6, while it was more similar with G3.2.6 in other regions than that with G3.14.1 (Supplementary Figure S6A). Furthermore, the epidemiological analysis showed that G3.2.6 and G3.14.1 were co-circulated and dominant in Japan for a long time, suggesting this recombination event likely occurred in Japan in July 2021.

The other recombination event involving the G3.2.6 and G3.14.1 groups is shown in Figure 3B and Supplementary Materials, which was intergenic recombination. EPI_ISL_5701780 that was sampled on 18 August 2021 from Japan had the co-mutations 241 T, 913 T, 3037 T, 3267 T, 5388A, 5,986 T, 6954 T in ORF1a gene, which were from G3.2.6, and had the co-mutations 14,408 T, 15451A, 16,466 T, 19220 T, 21618G, 21,846 T, 23403G, 23604G, 24410A, 25,469 T, 26767C, 27638C, 27,752 T, 27874 T, 28170 T, 28461G, 28,881 T, 28916 T, 29402 T, and 29,742 T, which were from G3.14.1. The co-mutations in the same group formed the block structure, indicating EPI_ISL_5701780 belonged to case three. As shown in Supplementary Figure S5B, its recombinant region and non-recombinant region inferred by RDP4 were 1 ~ 11,623 nt and 11,624 ~ end, respectively. The sequence similarity networks showed that EPI_ISL_5701780 was more similar with G3.2.6 between 1 and 11,623 nt in genomes than that with G3.14.1, while it was more similar with G3.14.1 in the rest region of genomes than that with G3.2.6 (Supplementary Figure S6B). In addition, the epidemiological analysis indicated that this recombination event likely occurred in Japan in August 2021.

The sources of gene fragments of the two recombinants are different, although both parents of them were G3.2.6 and G3.14.1. Notably, the spike protein of EPI_ISL_4697938 was from G3.2.6, inheriting the functional mutations N501Y and P681H. While the spike protein of EPI_ISL_5701780 came from G3.14.1, inheriting the functional mutations T478K and P681R. Beyond the acquisition of a set of functional spike mutations of G3.2.6 or G3.14.1, there are no obvious biological advantages that can be attributed to these recombinants.

### The Spatio-Temporal Distribution of Recombinants and Their Parental Groups

**Figure 4:**
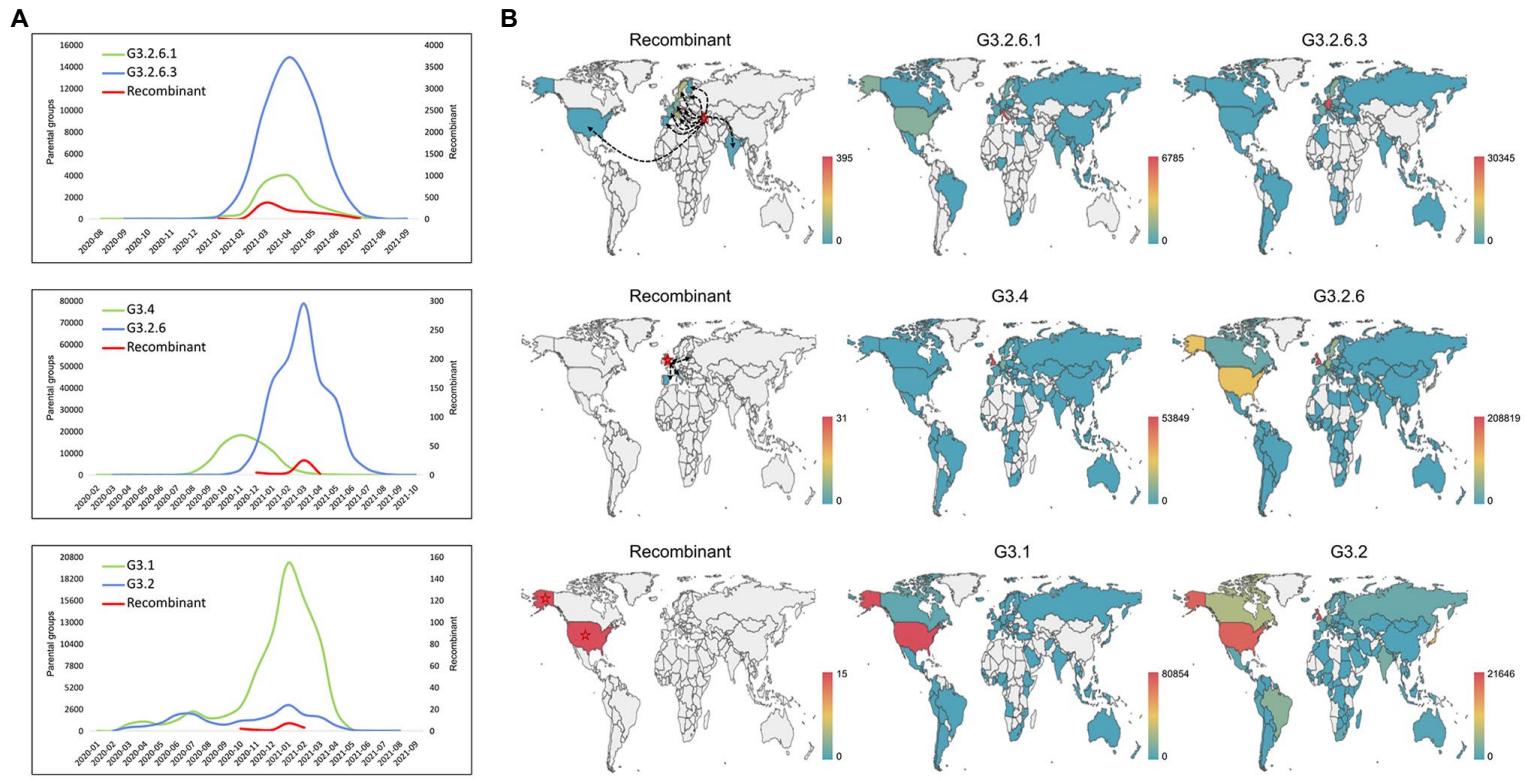
Spatio-temporal distribution of the recombinants and their parental groups. **(A)** The temporal distribution of the recombinant and its parents. Three representative recombination types are shown: G3.2.6.1 and G3.2.6.3, G3.4 and G3.2.6, and G3.1 and G3.2. **(B)** The spatial distribution of the recombinant and its parents. Three representative recombination types are shown: G3.2.6.1 and G3.2.6.3, G3.4 and G3.2.6, and G3.1 and G3.2. The red stars and the black dotted lines with arrows represent the country where the recombinant was collected for the first time, and the likely transmission direction of recombinants, respectively.

As shown in Figure 4A, we analyzed the temporal distribution of these recombination types, where three representative recombination types were shown. The recombination events hosted by G3.2.6.1 and G3.2.6.3 were earliest found in January 2021, which persisted at low frequency for 2 months before expanding and then decreasing. A similar distribution was observed in the other recombination types, indicating that the currently detected recombinants have not developed into the dominant variants. Compared with the temporal distributions of the recombinants, the parental variants kept co-circulation from the beginning to the end of the recombination event.

In addition, the spatial distributions of the three representative recombination types are shown in Figure 4B. The recombinants of G3.2.6.1 and G3.2.6.3 were earliest collected in Georgia and later sampled in Italy, France, Slovakia, Germany, Sweden et al. It is likely that the recombinants in other countries came from Georgia, but it is likely that they were caused by the co-infection of local people, as the parental groups also co-circulated in these countries. The recombinants evolved from G3.4 and G3.2.6 were collected in multiple countries, including the United Kingdom where the recombinant was collected for the first time, Latvia, Italy, Spain, and Denmark. Most of the recombinants were sampled in the United Kingdom where the parental groups G3.4 and G3.2.6 were dominant. In the recombination type hosted by G3.1 and G3.2, all the recombinants were sampled in the United States, which is the origin of most of the parental variants. These results showed that the parental groups co-circulated in the sampled regions of recombinants, and that the probability of recombination is high when the co-circulating variants were dominant in a region.

### Characteristics of Spike and Nucleoprotein Genes in Genetic Recombination

**Figure 5:**
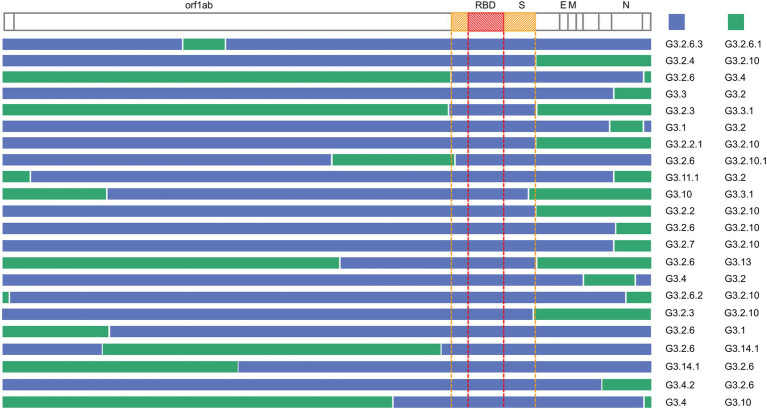
Summary of the recombination sites in the detected recombination types. Blocks matching the parental group contributing the S gene are shown in blue, while blocks matching other groups are colored in green. The region between the yellow dotted lines is the S gene, and the region between the red dotted lines is RBD.

The inferred recombination regions for some recombination types (the number of the recombinants was at least two) are shown in Figure 5. The inferred breakpoints in 82% (18/22) of recombination types were located in the 5′ or the 3′ of S and N genes, indicating that the 5′ or 3′ of S and N genes have high frequencies to form the recombination junctions. The recombinants often inherited the N gene from G3.2 or G3.2.10, the former containing the co-mutations G28881A, G28882A, and G28883C, the latter including A28877T, G28878C, G28881A, G28882A, and G28883C. The most frequent donor G3.2.6 usually contributed the functional spike protein which included the non-synonymous co-mutations N501Y, A570D, P681H, T716I, S982A, and D1118H. Interestingly, the RBD region of S gene was exchanged as a whole in all recombination types, which suggested that the RBD protein-coding region may be of a modular design to keep a robust exchange in SARS-CoV-2.

## Discussion

In this study, a method to identify the genetic events of SARS-CoV-2 variants was developed based on the classification of SARS-CoV-2 proposed in our previous work (Qin et al., 2021). Each group corresponds to a set of specific co-mutations that captured the vital evolutionary information of SARS-CoV-2 and the evolutionary relationship between groups accurately. Our classification clearly revealed the additive feature of co-mutation modules, and systematically reflected the evolution patterns of SARS-CoV-2. The classification proposed by the WHO mainly focuses on the epidemiology of SARS-CoV-2 variants, including the transmissibility, virulence, clinical representation, and so on. The WHO named the variants that significantly affected the current public health by integrating the nomenclature systems including GISAID (https://www.gisaid.org/), Nextstrain (https://nextstrain.org/sars-cov-2/) and Pango (Rambaut et al., 2020). It is easier to attract public attention and more practical to be discussed by non-scientific audiences but cannot reflect the evolution of SARS-CoV-2 effectively. On the other hand, our classification was based on the co-mutations, which avoided the statistical uncertainty and limitations in computation and visualization of the phylogenetic tree-based classification like GISAID, Nextstrain, and Pango.

We draw a complete genetic map showing the relationships among all SARS-CoV-2 variants, in which the parental groups of these variants and their evolutionary path were indicated. For emerging SARS-CoV-2 strains, the evolutionary path and its parental groups can be identified quickly based on our method. The construction of a high-quality genetic map was driven by intensive surveillance and large-scale sequencing. However, the sampling biases and incomplete information of the samples would influence the refinement of the genetic map. To address this issue, we can simulate the intermediate variants to connect the parents and descendants, based on the prior knowledge and viral evolution patterns. In addition, undetected infection paths among hosts can be inferred by combining intra-host genomic diversity with data-driven epidemiological models (Ramazzotti et al., 2021).

As of 31 October 2021, the genetic events detected in 3,392,553 SARS-CoV-2 genomes were mapped on the genetic map, which included 58 groups involving genetic mutation and 46 recombination types (a total of 1,229 recombinants). We validated the recombination events from three aspects: (i) the sequence similarity of each recombinant fragment with the inferred parents, (ii) the topology in phylogenetic trees of recombination region and non-recombination region, and (iii) the spatial–temporal distribution of the parental groups. Recombinations are caused by the viral polymerase of active replication jumping from one template to another when there is a co-infection of at least two genetically distinct genomes (Kirkegaard and Baltimore, 1986; Simon-Loriere et al., 2011). Although the co-circulation of parental viruses was observed which enabled co-infection (Sabir et al., 2016), more reliable methods should be developed to determine the co-infection of parental viruses. For example, a recent study by Zhou et al. captured the co-infection events in large-scale sequencing data and provided a framework for detecting the SARS-CoV-2 co-infection events in the Next-Generation Sequencing (NGS) data (Zhou et al., 2021).

For all detected recombination types, they did not show the advantage in community transmission, even the recombinants that inherited the spike gene with functional mutations from the dominant variants like G3.2.6 and G3.14.1. Taking the amino acid mutations in G3.2.6/Alpha as an example, N501Y increases the binding ability of RBD:ACE2, the infectivity, and the neutralization resistance (Supasa et al., 2021); P681H optimizes the cleavage of S protein by Flynn protease (Garcia-Beltran et al., 2021); and T716I contributes to the higher infectivity (Tian et al., 2021; Supplementary Table S2). This observation may be related to the modular transfer of RBD region. The mutations on RBD were not shuffled to obtain new mutation combinations, while the old mutation combinations kept dominant in the form of parental group. In addition, many studies have shown that spike domain exchange is an important evolutionary mechanism in the reported recombination events of many coronaviruses, which have been called “modular evolution” of the spike protein (Charlesworth et al., 2009; Graham and Baric, 2010; Vakulenko et al., 2021). The RBD of spike protein is generally the principal player in determining the host range, and the shuffle of various RBD moieties between virus strains may lead to host range expansion (Graham and Baric, 2010).

Genetic recombination not only plays an important role in the evolution of SARS-CoV-2 but also has a close relationship with its origin. Some studies showed that SARS-CoV-2 may originate from multiple recombination events (Boni et al., 2020; Domingo, 2021; Makarenkov et al., 2021). In the research of Makarenko et al., the evolutionary relationship between SARS-CoV-2 and 21 related coronaviruses was explored, which identified a detailed list of statistically significant horizontal gene transfer and recombination events for SARS-CoV-2 origin. We also analyzed the 21 related coronaviruses based on our developed method. The results showed that the genomes of RaTG13 and ZXC21 had partial co-mutations of G3.2.6.2, the GD Pangolin P2S and GD Pangolin 1 genomes had a part of co-mutations of G3.4, and the other 17 coronaviruses had some co-mutations of G3.2.10.1 in their genomes. The results indicated that these animal-origin coronaviruses may indeed donor their genomic materials to create the prototype SARS-CoV-2, which is consistent with the conclusion in previous studies. In addition, Makarenkov et al. revealed that the S and N genes of SARS-CoV-2 may result from intragenic recombination between RaTG13 and Guangdong (GD) Pangolin coronaviruses. Notably, our study found that the 5′ or 3′ of S and N genes had high frequencies to form the recombination junctions in the evolution of SARS-CoV-2, which was consistent with Gribble’s study (Gribble et al., 2021; Turkahia et al., 2021). These inspired we can focus on the S and N genes to explore the origin and evolution of SARS-CoV-2. Currently, with the co-circulation of multiple variants increasing the probability of recombination, close monitoring is needed to capture the novel recombination events, in particular, the recombinants with exchanged moieties on spike protein.

## Data Availability Statement

The SARS-CoV-2 genome sequences used in this paper are publicly available from the GISAID database (https://www.gisaid.org/). The information of SARS-CoV-2 grouping and the co-mutations used in our analysis is available on the website we developed: http://cmmgroup.grmh-gdl.cn:20023. The source codes for identifying the genetic events and drawing the spatial distribution map were released on GitHub (https://github.com/qinluoo/Genetic-events-of-SARS-CoV-2). The updated results as of 1st April 2022 were provided in the Supplementary Material.

## Author Contributions

TJ conceived and supervised the project. XD refined the framework and assisted with writing. LQ designed the workflow, performed computations, analyzed the results, and drafted the manuscript. All authors contributed to the article and approved the submitted version.

## Funding

This work was supported by the National Natural Science Foundation of China (32070678 and 31671371); the Emergency Key Program of Guangzhou Laboratory, grant no. EKPG21-12; the National Key Research and Development Program of China (2020YFC0840800); the CAMS Initiative for Innovative Medicine (CAMS-I2M and 2016-I2M-1-005).

## Conflict of Interest

The authors declare that the research was conducted in the absence of any commercial or financial relationships that could be construed as a potential conflict of interest.

## Publisher’s Note

All claims expressed in this article are solely those of the authors and do not necessarily represent those of their affiliated organizations, or those of the publisher, the editors and the reviewers. Any product that may be evaluated in this article, or claim that may be made by its manufacturer, is not guaranteed or endorsed by the publisher.

## Footnotes

^1^ https://bigd.big.ac.cn/ncov/variation/annotation

## Supplementary Material

Supplementary Table S1 Confirmation table of recombination events validated by different methods in RDP4 software.

Supplementary Table S2 Amino acid mutations of S protein in each group and the effect.

Supplementary Table S3 Sequences involving genetic recombination.
