# Supplementary material for: Mapping Genetic Events of SARS-CoV-2 Variants
Source: Front Microbiol. 2022 Jul 14;13:890590. doi: 10.3389/fmicb.2022.890590 (PMC9329953; doi:10.3389/fmicb.2022.890590)

Figure S1. Summary of genetic events in SARS-CoV-2. The detected genetic events and the number of samples contained are listed.

Belonging to a group			
G3.2.6	552606	G3.2.1	2565
G3	92232	G3.2.2	1997
G3.2	85407	G3.2.5	1970
G3.4	84211	G3.5	1705
G3.2.6.2	76136	G5	1657
G3.2.6.3	60451	G7	1581
G3.1	56750	G1.5	1222
G3.3	23751	G3.3.1	1212
G3.11.1	21393	G3.6	944
G3.2.6.2.1	18306	G6	872
G3.9.1	18141	G3.2.10	867
G3.2.4	16051	G1.1	853
G3.2.3	15249	G3.11	835
G3.4.3	13431	G3.2.8	779
G3.11.2	10418	G1	713
G3.2.7	8696	G1.2	631
G3.2.6.1	8590	G3.12	490
G3.4.2	6681	G3.10	473
G3.4.1	6185	G3.14.1.2	400
G0	6059	G3.13.1	381
G3.2.2.1	5812	G3.14.1.1	335
G3.14	5745	G3.13	333
G3.1.1	4707	G3.14.1.3	321
G3.4.4	4671	G3.2.9	229
G3.9	4287	G1.3	140
G3.8	4247	G4	68
G3.7	4093	G1.4	59
G3.14.1	3597	G2	34
G3.2.10.1	3403	G2.1	11
G1.6	2690		

0 560000

Genetic mutation invoving a group			
G3.14.1	1164957	G3.2.4	2263
G3.14	218906	G3.11	2184
G3.2.6	170082	G1	1831
G3.14.1.1	85405	G1.5	1662
G3.14.1.2	84771	G3.11.2	1637
G3.2.10.1	80141	G3.4.3	1429
G3.14.1.3	61082	G3.4.2	1341
G3.1	30044	G3.9	1031
G3.3.1	29405	G3.4.1	963
G3.13.1	23662	G3.5	858
G3.12	22081	G1.2	677
G3.2	19094	G3.7	623
G3.2.9	19044	G3.4.4	585
G3.2.6.2	14445	G3.2.7	556
G3.4	14115	G3.2.1	478
G3.10	12849	G2	355
G3.3	12792	G3.2.2	214
G3.2.6.3	9883	G3.8	210
G3.13	9573	G2.1	195
G3.11.1	6208	G3.2.10	194
G3.2.8	5521	G7	151
G3.2.2.1	4836	G5	144
G3.1.1	4678	G3.6	140
G3	4582	G1.6	122
G3.2.5	4295	G1.1	90
G3.2.6.1	3389	G6	25
G3.2.6.2.1	2783	G1.4	9
G3.9.1	2594	G1.3	5
G3.2.3	2462		

0 1200000

Genetic recombination involving two groups			
G3.2.6.3&G3.2.6.1	833	G3.10&G3.2	1
G3.2.4&G3.2.10	228	G3.2.6&G3.4	1
G3.4&G3.2.6	37	G3.2.6&G3.2.10.1	1
G3.3&G3.2	18	G3.2.6&G3.1	1
G3.3.1&G3.2.3	17	G3.3.1&G3.2.6	1
G3.1&G3.2	15	G3.2.6.2.1&G3.2.10	1
G3.2.2.1&G3.2.10	7	G3.9.1&G3.2.6	1
G3.2.10.1&G3.2.6	6	G3.11.1&G3.2.6	1
G3.11.1&G3.2	6	G3&G1.1	1
G3.10&G3.3.1	4	G3.2.5&G3.2.10	1
G3.2.6&G3.2.10	4	G3.1&G3.11.1	1
G3.2.7&G3.2.10	4	G3.2.6&G3.11.1	1
G3.2.2&G3.2.10	4	G3.4.2&G3.4.1	1
G3.4&G3.2	3	G3.13.1&G3.2	1
G3.13&G3.2.6	3	G3&G1.6	1
G3.2.6.2&G3.2.10	3	G3.11.2&G3.2.6	1
G3.2.3&G3.2.10	3	G3.2.6&G3.13	1
G3.2.6&G3.14.1	2	G3.2.6.2&G3.2.6.3	1
G3.4&G3.10	2	G3.4&G3.1	1
G3.14&G3.2.6	2	G3.10&G3.2.6	1
G3.4.2&G3.2.6	2	G3.11.1&G3.11.2	1
G3.1&G3.2.6	2	G3.12&G3.2	1
G3.3.1&G3.4	1	G3.2.6.2&G3.14	1

0 850

Figure S2. The phylogenetic trees of the recombination events hosted by G3.2.6/Alpha and G3.14.1/Delta. (A) Genetic recombination in EPI_ISL_4697938. (B) Genetic recombination in EPI_ISL_5701780.

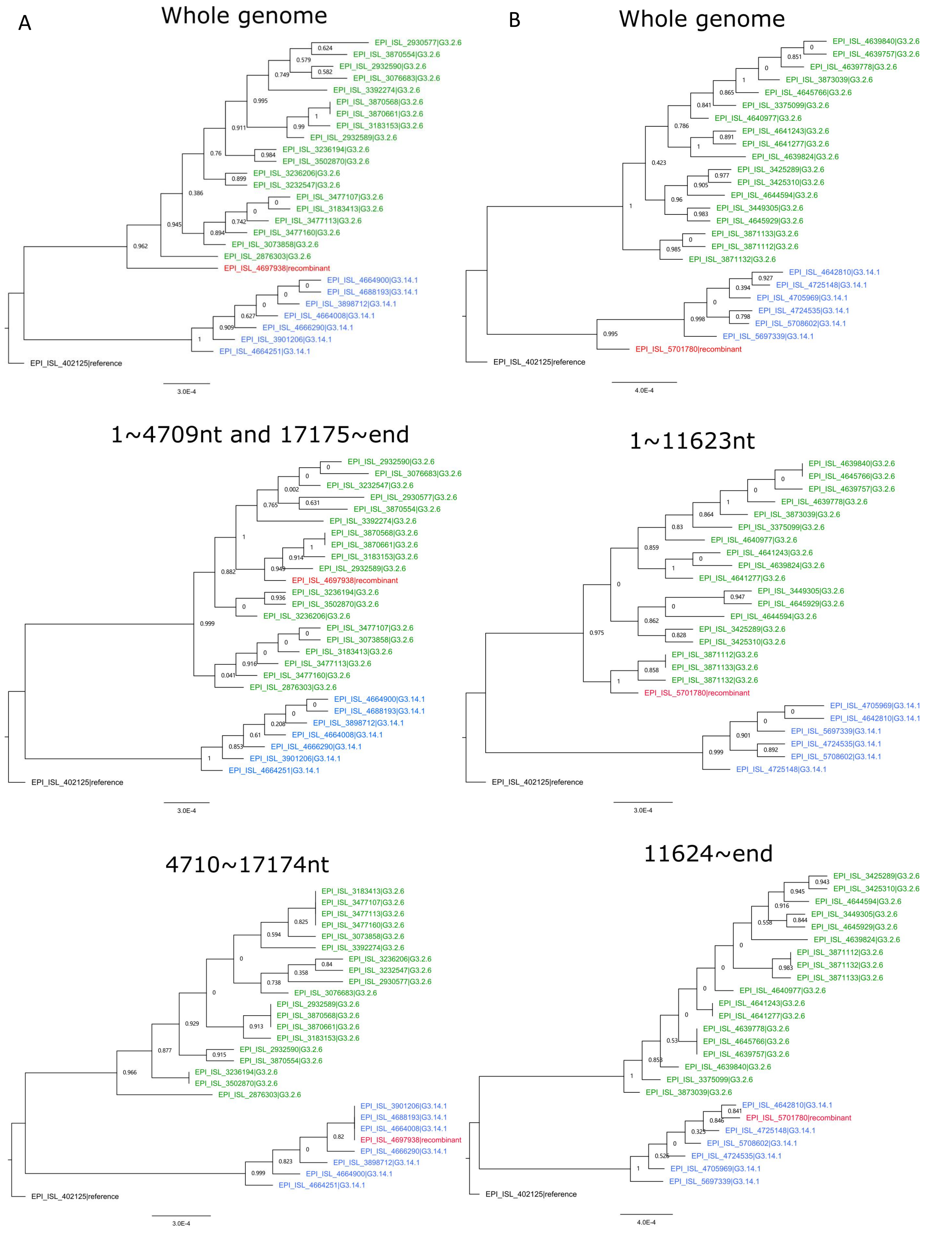

Figure S3. The whole genome trees and the split trees. The sequence with the least number of ambiguous bases and collected earliest in each group was selected to construct the phylogenetic trees, in which G0 was the original strain. (A) Phylogenetic trees of EPI_ISL_4697938 that was recombined from G3.2.6 and G3.14.1. (B) Phylogenetic trees of EPI_ISL_5701780 that was recombined from G3.2.6 and G3.14.1.

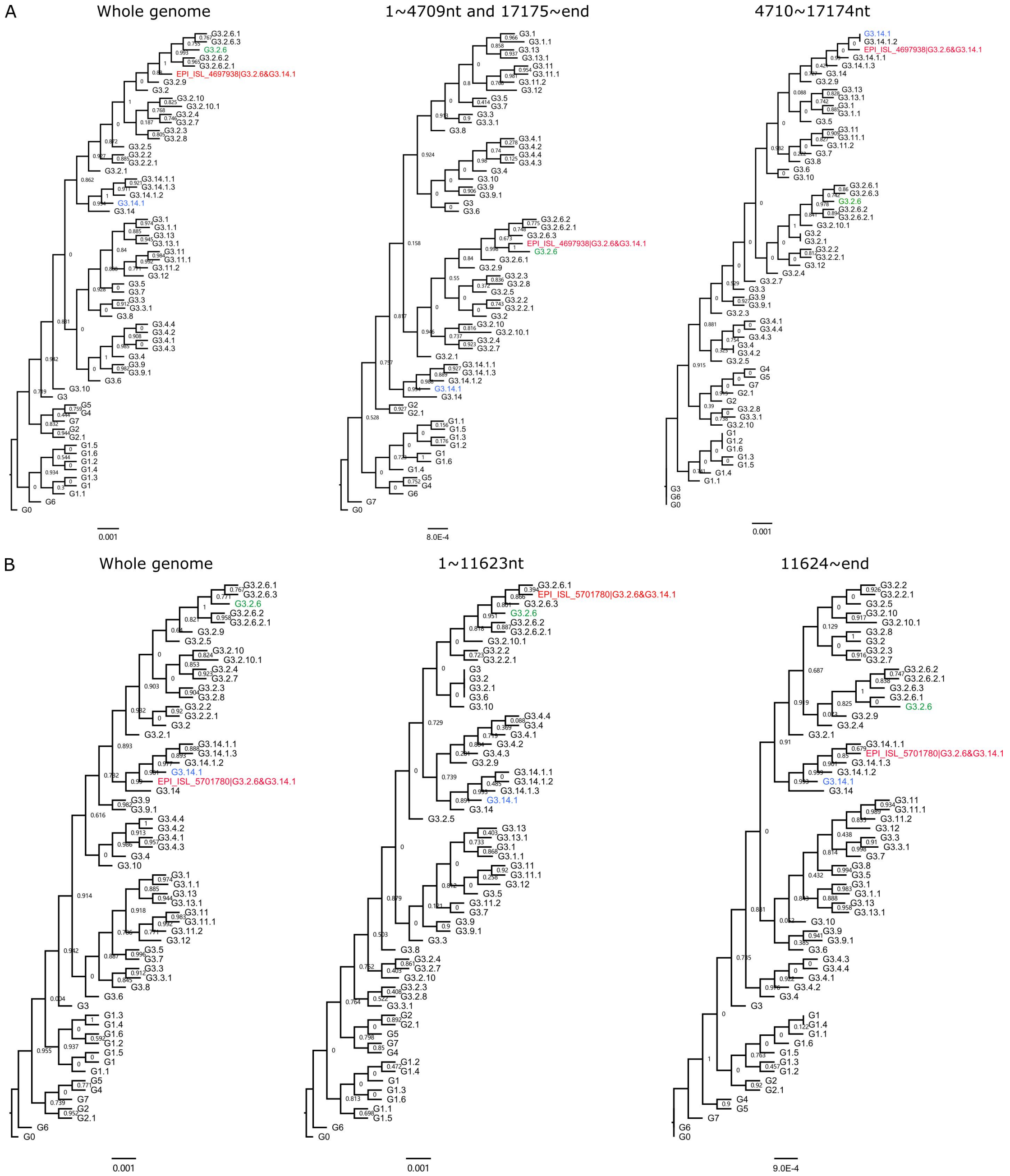

Figure S4. The Simplot analysis results of EPI_ISL_4697938 (A) and EPI_ISL_5701780 (B). The breakpoint positions were marked by the red dotted lines.

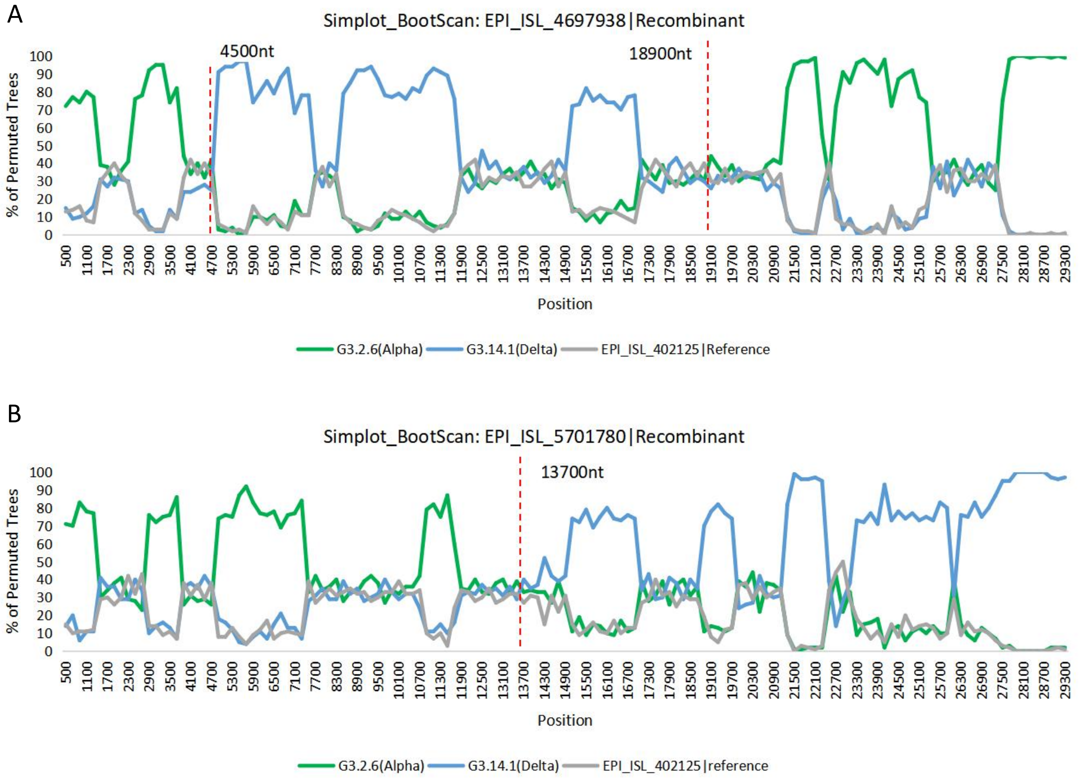

Figure S5. The RDP4 analysis results of EPI_ISL_4697938 (A) and EPI_ISL_5701780 (B).

A

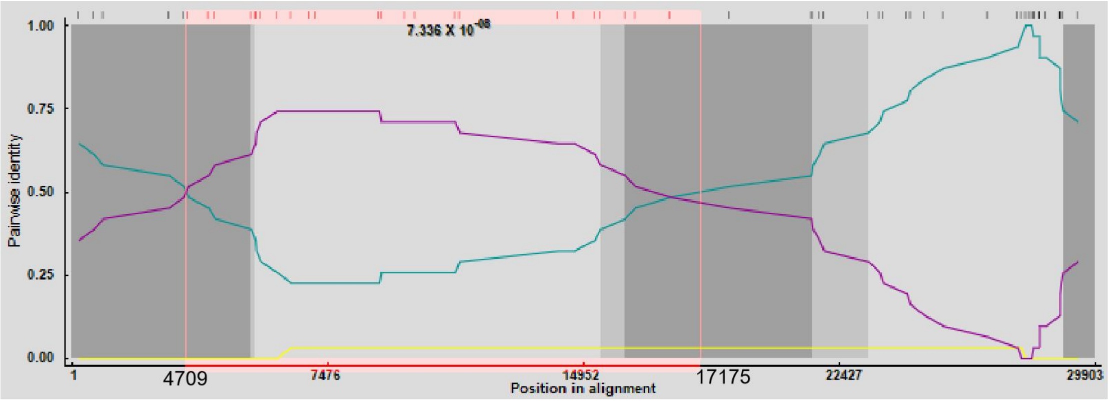

G3.2.6-G3.14.1 [Major Parent-Minor Parent]

G3.2.6-EPI_ISL_4697938 [Major Parent-Recombinant]

G3.14.1-EPI_ISL_4697938 [Minor Parent-Recombinant]

B

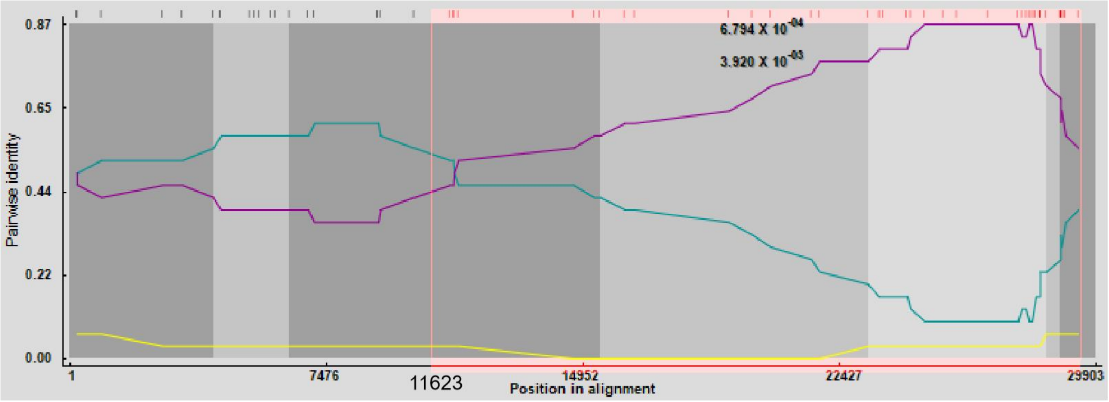

G3.2.6-G3.14.1 [Major Parent-Minor Parent]

G3.2.6-EPI_ISL_5701780 [Major Parent-Recombinant]

G3.14.1-EPI_ISL_5701780 [Minor Parent-Recombinant]

Figure S6. The sequence similarity network and bootscan by Simplot++ of EPI_ISL_4697938 (A) and EPI_ISL_5701780 (B). The more the red dots in the sequence similarity network, the higher the similarity.

A

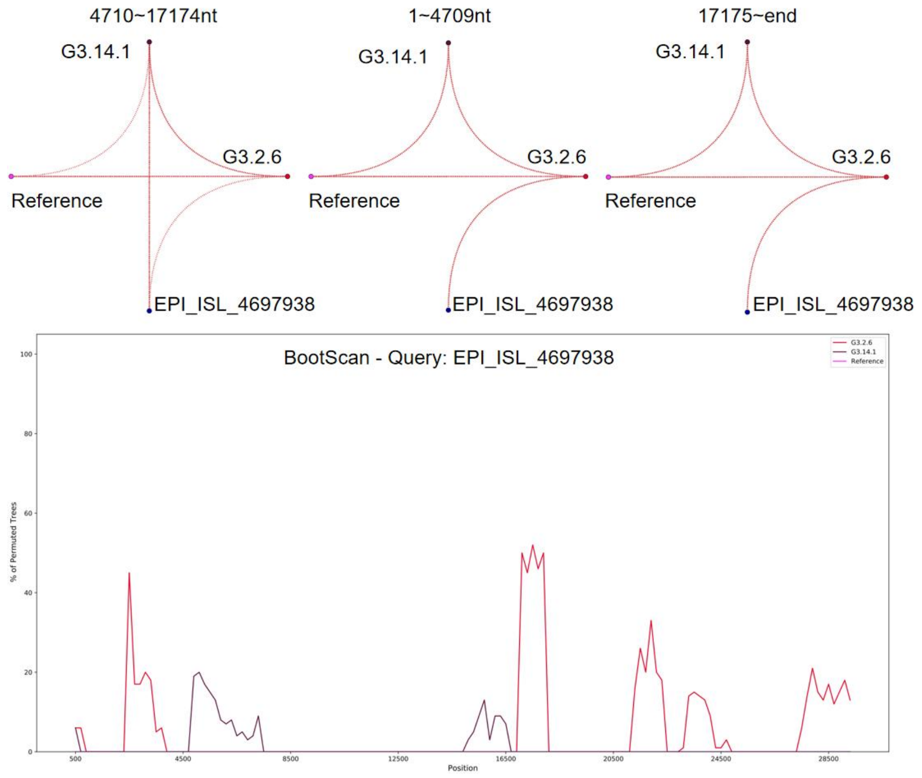

B

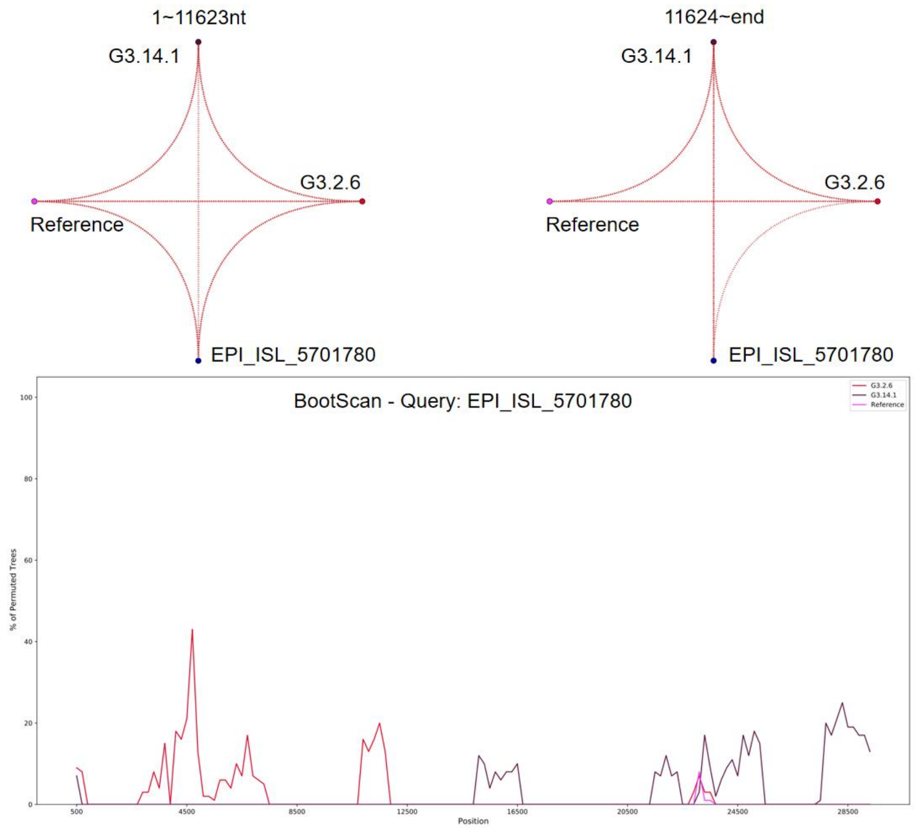

Supplement: Supplementary file 2 [file Data_Sheet_1.PDF]
