# Supplementary material for: Mapping Genetic Events of SARS-CoV-2 Variants
Source: Front Microbiol. 2022 Jul 14;13:890590. doi: 10.3389/fmicb.2022.890590 (PMC9329953; doi:10.3389/fmicb.2022.890590)
Supplement: Supplementary file 3 [file Data_Sheet_2.PDF]

## The updated data and results as of 1st April 2022 were shown below.

**Data collection and processing**

A total of 5,094,025 SARS-CoV-2 high-quality genomes sampled from humans were downloaded from the GISAD database as of 1st April 2022 with the labels of "complete", "high coverage" and "collection date complete". 5,000,442 sequences submitted to GISAD as of 1st March 2022 were used to identify the co-mutations. After the data processing using the pipeline described in previous work, the remaining 3,989,533 genomes were used for the genetic grouping. The 5,094,025 genomes were aligned to the SARS-CoV-2 reference genome in GenBank (NCBI Accession number: NC_045512.2) using MAFFT.

**Three new groups**

In the updated result, three new groups were identified, including G3.2.11, G3.2.11.1, and G3.2.11.1.1, which were corresponding to the Omicron variant. The co-mutations in each group were shown below.

G3.2.11:

23055_G,26577_G,27807_T,27259_C,23075_C,22674_T,22679_C,22578_A,23040_G,24469_A,18163_G,23599_G,22686_T,26709_A,24424_T,10449_A,23854_A

G3.2.11.1:

27383_T,27382_C

G3.2.11.1.1:

670_G,2790_T,22786_C,9534_T,22200_G,29510_C,9424_G,22688_G,10447_A,22775_A,9866_T,4184_A

**91 new recombinants**

| | | | |
|---|---|---|---|
| EPI_ISL_9034243 | G3.2.6.3&G3.2.6.1 | EPI_ISL_11229604 | G3.3.1&G3.9.1 |
| EPI_ISL_8385799 | G3.2.6.3&G3.2.6.1 | EPI_ISL_11229880 | G3.3.1&G3.9.1 |
| EPI_ISL_8385819 | G3.2.6.3&G3.2.6.1 | EPI_ISL_11229953 | G3.3.1&G3.9.1 |
| EPI_ISL_8402903 | G3.2.6.3&G3.2.6.1 | EPI_ISL_11229892 | G3.4&G3.9.1 |
| EPI_ISL_8403137 | G3.2.6.3&G3.2.6.1 | EPI_ISL_11229626 | G3.9&G3.4 |
| EPI_ISL_8402894 | G3.2.6.3&G3.2.6.1 | EPI_ISL_11229946 | G3.3.1&G3.9.1 |
| EPI_ISL_8403142 | G3.2.6.3&G3.2.6.1 | EPI_ISL_11229094 | G3.9&G3.2.6 |
| EPI_ISL_8438589 | G3.2.6.3&G3.2.6.1 | EPI_ISL_11229134 | G3.3.1&G3.9.1 |
| EPI_ISL_8438559 | G3.2.6.3&G3.2.6.1 | EPI_ISL_11229310 | G3.4&G3.9.1 |
| EPI_ISL_8438566 | G3.2.6.3&G3.2.6.1 | EPI_ISL_11229388 | G3.9&G3.2.6 |
| EPI_ISL_8438672 | G3.2.6.3&G3.2.6.1 | EPI_ISL_11229308 | G3.3.1&G3.9.1 |
| EPI_ISL_8438669 | G3.2.6.3&G3.2.6.1 | EPI_ISL_11229317 | G3.4&G3.9.1 |
| EPI_ISL_8438660 | G3.2.6.3&G3.2.6.1 | EPI_ISL_11229548 | G3.4&G3.9 |
| EPI_ISL_8438620 | G3.2.6.3&G3.2.6.1 | EPI_ISL_11229554 | G3.4&G3.9.1 |
| EPI_ISL_8438637 | G3.2.6.3&G3.2.6.1 | EPI_ISL_11229555 | G3.9&G3.4 |
| EPI_ISL_8438691 | G3.2.6.3&G3.2.6.1 | EPI_ISL_11229552 | G3.9&G3.4 |

| | | | |
|---|---|---|---|
| EPI_ISL_8438642 | G3.2.6.3&G3.2.6.1 | EPI_ISL_11229561 | G3.3.1&G3.9.1 |
| EPI_ISL_8438655 | G3.2.6.3&G3.2.6.1 | EPI_ISL_11229671 | G3.4&G3.2.6 |
| EPI_ISL_8438587 | G3.2.6.3&G3.2.6.1 | EPI_ISL_11229676 | G3.9&G3.2.6 |
| EPI_ISL_8438623 | G3.2.6.3&G3.2.6.1 | EPI_ISL_11229637 | G3.3.1&G3.9.1 |
| EPI_ISL_8438686 | G3.2.6.3&G3.2.6.1 | EPI_ISL_11229636 | G3.9&G3.4 |
| EPI_ISL_8438659 | G3.2.6.3&G3.2.6.1 | EPI_ISL_11229372 | G3.9&G3.2.6 |
| EPI_ISL_8438576 | G3.2.6.3&G3.2.6.1 | EPI_ISL_11229589 | G3.3.1&G3.9.1 |
| EPI_ISL_8438622 | G3.2.6.3&G3.2.6.1 | EPI_ISL_11229492 | G3.9&G3.4 |
| EPI_ISL_8438650 | G3.2.6.3&G3.2.6.1 | EPI_ISL_11229292 | G3.9&G3.2.6 |
| EPI_ISL_8438649 | G3.2.6.3&G3.2.6.1 | EPI_ISL_11229242 | G3.4&G3.9.1 |
| EPI_ISL_8501646 | G3.2.6.3&G3.2.6.1 | EPI_ISL_11229294 | G3.9&G3.2.6 |
| EPI_ISL_8501740 | G3.2.6.3&G3.2.6.1 | EPI_ISL_11229293 | G3.9&G3.2.6 |
| EPI_ISL_8501729 | G3.2.6.3&G3.2.6.1 | EPI_ISL_11229319 | G3.3.1&G3.9.1 |
| EPI_ISL_8502938 | G3.2.6.3&G3.2.6.1 | EPI_ISL_11229673 | G3.2.6&G3.4 |
| EPI_ISL_8502964 | G3.2.6.3&G3.2.6.1 | EPI_ISL_11229524 | G3.4&G3.9.1 |
| EPI_ISL_8502932 | G3.2.6.3&G3.2.6.1 | EPI_ISL_11229608 | G3.9&G3.4 |
| EPI_ISL_8502936 | G3.2.6.3&G3.2.6.1 | EPI_ISL_11229609 | G3.3.1&G3.9.1 |
| EPI_ISL_10341952 | G3.2.4&G3.2.10 | EPI_ISL_11229621 | G3.4&G3.9.1 |
| EPI_ISL_10341881 | G3.2.4&G3.2.10 | EPI_ISL_11229969 | G3.9&G3.4 |
| EPI_ISL_10342015 | G3.2.4&G3.2.10 | EPI_ISL_11229279 | G3.3.1&G3.9.1 |
| EPI_ISL_10341911 | G3.2.4&G3.2.10 | EPI_ISL_11230330 | G3.9&G3.4 |
| EPI_ISL_10342543 | G3.2.4&G3.2.10 | EPI_ISL_11230341 | G3.9&G3.4.3 |
| EPI_ISL_9467354 | G3.2.6.3&G3.2.6.1 | EPI_ISL_11230328 | G3.4&G3.2.6 |
| EPI_ISL_9467386 | G3.2.6.3&G3.2.6.1 | EPI_ISL_7857911 | G3.1&G3.2 |
| EPI_ISL_9467387 | G3.2.6.3&G3.2.6.1 | EPI_ISL_7727302 | G3.2.6&G3.3.1 |
| EPI_ISL_11229876 | G3.4&G3.9.1 | | |
